# Initiation of DNA Replication from Non-Canonical Sites on an Origin-Depleted Chromosome

**DOI:** 10.1371/journal.pone.0114545

**Published:** 2014-12-08

**Authors:** Naomi L. Bogenschutz, Jairo Rodriguez, Toshio Tsukiyama

**Affiliations:** 1 Basic Sciences Division, Fred Hutchinson Cancer Research Center, Seattle, Washington, United States of America; 2 Molecular and Cellular Biology Program, University of Washington and Fred Hutchinson Cancer Research Center, Seattle, Washington, United States of America; University of Oxford, United Kingdom

## Abstract

Eukaryotic DNA replication initiates from multiple sites on each chromosome called replication origins (origins). In the budding yeast *Saccharomyces cerevisiae*, origins are defined at discrete sites. Regular spacing and diverse firing characteristics of origins are thought to be required for efficient completion of replication, especially in the presence of replication stress. However, a *S. cerevisiae* chromosome III harboring multiple origin deletions has been reported to replicate relatively normally, and yet how an origin-deficient chromosome could accomplish successful replication remains unknown. To address this issue, we deleted seven well-characterized origins from chromosome VI, and found that these deletions do not cause gross growth defects even in the presence of replication inhibitors. We demonstrated that the origin deletions do cause a strong decrease in the binding of the origin recognition complex. Unexpectedly, replication profiling of this chromosome showed that DNA replication initiates from non-canonical loci around deleted origins in yeast. These results suggest that replication initiation can be unexpectedly flexible in this organism.

## Introduction

DNA replication in eukaryotic cells initiates from multiple sites on each chromosome called replication origins (hereafter origins). In the budding yeast *Saccharomyces cerevisiae*, origins contain defined discrete DNA sequences termed autonomously replicating sequences (ARSs), so-named because of their ability to maintain plasmid propagation [1]. The existence of well-defined origins in *S. cerevisiae* have enabled extensive origin mapping and characterization studies by many researchers, which have led to a detailed understanding of the replication landscape in budding yeast [2]–[5]. These studies have revealed that between late G2/M and late G1 phases of the cell cycle, multiple proteins are targeted to origins in an ordered fashion to form pre-replicative complexes (pre-RCs). The formation of a pre-RC on an origin makes it competent to initiate replication (fire) during the following S phase [6], [7]. The six subunit Origin Recognition Complex (ORC) is central to this process, as it directly binds to the ACS (ARS consensus sequence) within origins and recruits subsequent proteins for pre-RC formation [6].

Although pre-RCs are formed at all origins that have the ability to fire in the following S phase, each origin exhibits different characteristics in timing (when in S phase the origin fires) and efficiency (how likely in a given S phase the origin fires). It is generally believed that these properties, along with proper origin spacing, are essential for chromosome stability, as dormant origins (late-firing, inefficient origins) can act as backups to early-firing, efficient origins if replication forks collapse in early S-phase [8], [9]. Indeed, dormant origins are activated when efficient origins are deleted on an extra copy of chromosome III [10]. Interestingly, this chromosome harboring deletions of several active and dormant origins was still stably maintained, despite an elevated loss rate compared to a wild-type chromosome III [10]. The replication checkpoint protein Rad9 and components of the DNA damage repair pathway have been shown to be important in propagating this and other origin deficient chromosomes [11]–[13]. These results argue that *S. cerevisiae* chromosome III with a greatly reduced number of canonical origins can be replicated relatively normally. However, how replication of the origin-deficient chromosome is accomplished is not understood.

In this study, we aimed to establish an independent system to investigate how an origin-deficient chromosome is replicated by systematically deleting seven well-characterized origins on the left arm of *S. cerevisiae* chromosome VI. We found that the strain exhibits no detectable growth defects, even in the presence of DNA damaging agents and replication inhibitors. Unexpectedly, we detected initiation of DNA replication from loci around deleted origins, despite the fact that origin deletions cause sharp decrease in ORC binding at deleted origins. Our results suggest that initiation of DNA replication can be unexpectedly flexible in *S. cerevisiae*, an organism that normally initiates DNA replication from discrete sites.

## Materials and Methods

### Yeast strains

Strains used in this study were created using standard yeast manipulations and are listed in Table 1. Cells were grown in standard YPD medium or synthetic selective medium as appropriate. All strains were derived from W303-1a strain in which a weak *rad5* mutation in the original W303-1a was corrected [14], [15]. The *7ori*Δ strain was constructed by deleting confirmed ARS sequences identified by [16], the Origin Database (OriDb) [5] and on the Saccharomyces Genome Database (www.yeastgenome.org). The exact regions of deletion are listed in Table 2. Origin deletion was done in the following order by one step replacement: *ARS600::LEU2, ARS606::ADE2, ARS601, 602::loxP, ARS605::loxP, ARS603::loxP, ARS604::loxP, ARS603.5::KanMX*. The loxP alleles were created by looping out the KanMX cassette using the Cre-lox system as described [17]. Each deletion was confirmed by PCR using primers that do not overlap with the oligonucleotides used for deletions. We confirmed that two completely independently created *7ori*Δ strains exhibit the same growth phenotypes. In addition, through a large number of genetic crosses, we confirmed that the deleted origins co-segregate at an expected rate.

**Table 1:** Strains used in this study.

Strain	Genotype	Reference
W1588-4C	*MATa ade2-1 can1-100 his3-11,15 leu2-3,112 trp1-1 ura3-1 RAD5+*	[15]
YTT4024	*MATa ars600*Δ*::LEU2 ars601/2*Δ*::loxP ars603*Δ*::loxP ars603.5*Δ*::KAN ars604*Δ*::loxP ars605*Δ*::loxP ars606*Δ*::ADE2*	This study
YTT4977	*MATa mec1*Δ*::KAN sml1*Δ*::NAT*	This study
YTT5158	*MATa Orc2-2XLinker-3XFlag-klTRP1*	This study
YTT5160	*MATa Orc2-2XLinker-3XFlag-klTRP1 ars600*Δ*::LEU2 ars601/2*Δ*::loxP ars603*Δ*::loxP ars603.5*Δ*::KAN ars604*Δ*::loxP ars605*Δ*::loxP ars606*Δ*::ADE2*	This study
YTT5260	*MATa URA3::pRS306-BrdUinc ADE2+ LEU2+*	This study
YTT5262	*MATa URA3::pRS306-BrdUinc ars600*Δ*::LEU2 ars601/2*Δ*::loxP ars603*Δ*::loxP ars603.5*Δ*::KAN ars604*Δ*::loxP ars605*Δ*::loxP ars606*Δ*::ADE2*	This study

**Table 2:** *7ori*Δ origin deletions.

Origin	Replacement sequence	Chr VI coordinates deleted	Total base pairs deleted
ARS600	*LEU2*	5435–20,826	15,292
ARS601/2	loxP	32,473–33,247	775
ARS603	loxP	68,693–68,871	179
ARS603.5	*KanMX*	118,637–118,957	321
ARS604	loxP	127,751–128,071	321
ARS605	loxP	135,985–136,085	101
ARS606	*ADE2*	167,614–168,048	434

### ORC2 IP

Orc-bound DNA was isolated from Orc2-2Xlinker-3XFlag epitope-tagged cells arrested with nocodazole (15 µg/ml) for 2 hours at 30°C. Samples were cross-linked with 1% formaldehyde for 20 min at room temperature, and cross-lining was stopped with 0.125M glycine. Chromatin was sonicated using a Bioruptor sonicator bath (Diagenode), to an average fragment size of 250 bp. Soluble chromatin was immunoprecipitated as previously described [18] using antibodies against Flag (Sigma). Immunoprecipitation efficiency was checked by Western blot. Immunoprecipitated and input DNA were amplified using whole-genome amplification (Genome-Plex, Sigma-Aldrich).

### BrdU IP

DNA was harvested from cells harboring a single integrated BrdU incorporation vector [19] and replicated DNA was immunoprecipitated as described [20] with the following modifications: Lysis buffer contained 100 mM Tris-HCl pH 8.0 and 20% glycerol, cell lysate was sonicated using a Bioruptor sonicator bath (Diagenode), and BrdU immunoprecipitation was performed using Dynabeads Protein G (Novex). Input (G1 phase) and immunoprecipitated S-phase DNA was amplified using whole genome amplification (GenomePlex, Sigma-Aldrich).

### Tiling array hybridization and data processing

Enriched and input DNA was UDG fragmented and terminally labeled with Cy5 and Cy3, respectively, and competitively hybridized to custom tiling arrays (Roche NimbleGen) as previously described [21]. Arrays contain 50-mer probes overlapping by an average of 42 bp, covering both strands of chromosomes III, VI, and XII, representing 14% of the yeast genome. Nimblescan software was used to obtain Tukey by-weight mean-adjusted log2 Cy5/Cy3 ratio files, and a 50 base-pair pseudomedian sliding window was applied to all files to eliminate outliers [22], [23]. To allow for direct peak-height comparison between BrdU chromatin immuno-precipitation on microarrays (ChIP-Chip) samples, each data set was normalized by subtracting the average signal of known unreplicated regions in 200 mM hydroxyurea (HU) within each data set [24]. Known unreplicated regions included ChrIII 184–192 kb, ChrVI 80–86 kb, and ChrXII 845–870 kb. Accession number of the replication profile is GSE61743.

## Results and Discussion

### An origin deficient chromosome displays robust growth

**Figure 1:**
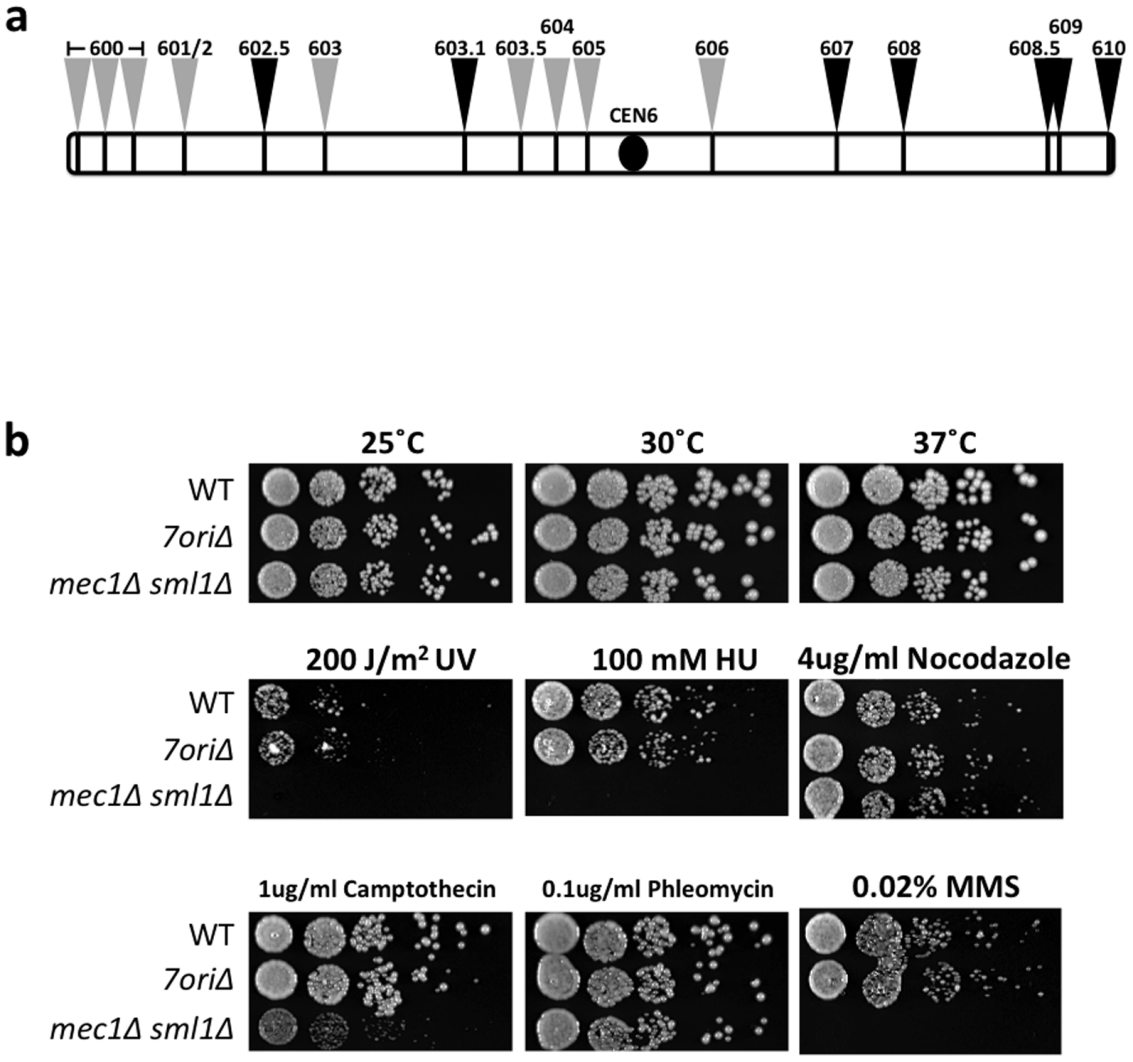
Chromosome VI *7ori*Δ ARS deletion strain does not exhibit gross growth defects even in the presence of replication stress. (a) A schematic drawing of ARS locations on chromosome VI. ARS sequence positions are represented by a black bar and labeled with an arrow. The centromere is denoted by a black circle. Deleted ARSs on the *7ori*Δ chromosome VI are labeled with grey arrows and ARSs that remained intact are labeled with black arrows. (b) Five-fold serial dilution assay of wild-type and 7*ori*Δ on indicated media. The *mec1*Δ* sml1*Δ S-phase checkpoint mutant is shown as a replication inhibitor positive control.

To establish a system to investigate how an origin-deficient chromosome can be replicated, we created the *7ori*Δ strain, a yeast mutant in which seven annotated and confirmed origins on the left arm of chromosome VI were deleted (Table 2; Fig. 1a). Although the effects of systematic origin deletion have been assessed using chromosome III [10], [12], [13], [25], the deletions were made in a background that carries an extra copy of chromosome III such that the mutant chromosome can be lost. Because the presence of an extra copy of a single chromosome can cause genomic instability [26], we wished to create a system in which origins are deleted without an extra copy of a chromosome. In addition, we sought to establish an independent system that does not utilize chromosome III, to alleviate possible chromosome-specific effects. Chromosome VI was chosen because origins and replication profiles of this chromosome have been well characterized [16]. In addition to origin deletions, the *7ori*Δ strain also possesses partial deletions of the non-essential proteasome activator *BLM10* and the meiotic recombination gene *MSH4* due to overlaps with ARS604 and ARS605, respectively. We first tested whether the *7ori*Δ strain displays growth defects because of the severe reduction in replication initiation events within the left arm of chromosome VI, especially when grown in the presence of replication inhibitors. Spot tests revealed that the *7ori*Δ strain exhibits no detectable growth defects on either rich (Fig. 1b) or synthetic (data not shown) media, consistent with reports on origin-deficient chromosome III [10], [11]. To test the model in which the presence of multiple functional origins is important under DNA damage and replication stress conditions, we tested the growth of the *7ori*Δ strain in the presence of HU and various DNA damaging conditions (Fig. 1b). Unexpectedly, deletion of 7 origins did not detectably affect cell growth even under these conditions. Indeed, we detected no measurable decrease in viability of the *7ori*Δ strain in the presence of HU or MMS (data not shown).

An extra copy of origin-depleted chromosome III has been reported to exhibit increased chromosome loss rates when combined with mutations in replication checkpoint and DNA damage pathway genes, including *RAD9* [11]–[13]. We tested the *7ori*Δ strain for interaction with genes known to contribute to these and other pathways. Interestingly, we found no genetic interaction with DNA damage pathway genes including *RAD9, MEC1*, and *RAD53*; the S-phase checkpoint genes *MRC1, TOF1*, and *RRM3*; and other cell cycle genes including *CLB5, MAD3*, and SIC1, even in the presence of DNA damaging agents (data not shown). This is likely partly due to the fact that the chromosome III loss assay in the presence of an extra copy of the chromosome is more sensitive than growth assays. We also cannot exclude the possibility that the effects of origin depletion are somewhat different between chromosomes III and VI, and/or that the presence of an extra copy of chromosome III sensitizes the system.

### Origin deletions cause reduced ORC binding

**Figure 2:**
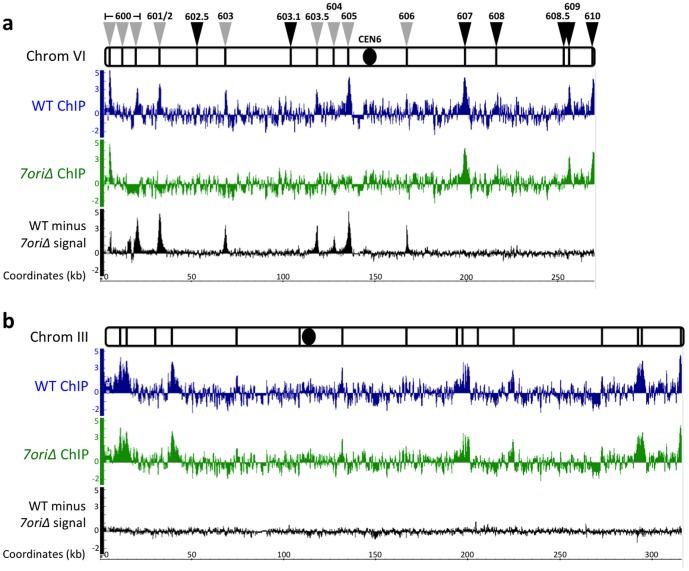
Deleted origins do not recruit Orc2. Orc2-3XFlag chromatin ChIP-Chip of (a) chromosome VI and (b) chromosome III (control) in G2/M arrested cells. Wild-type (blue) and *7ori*Δ (green). ARS positions are shown as black bars above each panel. The centromere is denoted by a black circle. Orc2 ChIP signals are shown, along with the difference between the wild-type and *7ori*Δ signal (black) to show level of Orc2 depletion in *7ori*Δ. The Y-axes show Orc2 signal ratio in log_2_ scale, and the X-axes show chromosomal coordinates.

To determine how the *7ori*Δ chromosome is replicated, we first measured how origin deletion affected the binding of the ORC across the left arm of chromosome VI, as the deleted sites have been confirmed to be essential for ORC binding and origin function [6]. To this end, we performed chromatin immuno-precipitation on high-density tiled microarrays (ChIP-Chip) of Orc2, one of the six subunits of ORC. As shown in Fig. 2, peaks of Orc2 signals are evident at most of the known origins in wild-type cells as expected. To visualize the effects of the *7ori*Δ mutation on ORC binding, we subtracted Orc2 signals in the *7ori*Δ strain from those in wild-type cells. This resulted in clear Orc2 peaks at every deleted origin on chromosome VI (Fig. 2a, black), demonstrating that the *7ori*Δ mutation did strongly reduce ORC binding at every deleted origin. In contrast, on chromosome III, where origins were not deleted, there was no notable difference in Orc2 signals between wild-type and *7ori*Δ cells (Fig. 2b). ORC ChIP signals are typically not as sharp and distinct as those of transcription factors. In addition, there are recent reports showing that ChIP data should be carefully interpreted [27]–[29]. However, we believe our Orc2 ChIP data faithfully reflects ORC binding *in vivo* because of sharp reduction of the signals specifically at deleted origins, and virtually identical signals on chromosome III. Therefore, these results showed that the *7ori*Δ strain exhibits robust growth that is indistinguishable from wild-type cells – even in the presence of replication inhibitors – despite the fact that it has strongly reduced levels of ORC binding across the left arm of chromosome VI.

### Replication initiates from non-canonical sites around deleted origins

**Figure 3:**
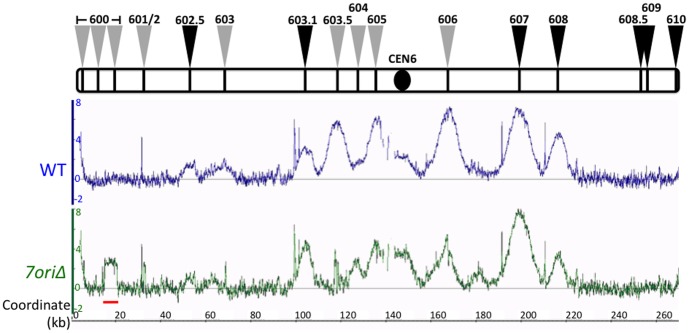
Non-canonical origins arise around deleted ARS sites. BrdU IP-ChIP replication profiles of chromosome VI. Wild-type (blue) and *7ori*Δ (green) cells were arrested in G1 and released into media containing BrdU and 200 mM HU for 60 min. The Y-axes show BrdU signal ratio in log_2_ scale using a non-replicated sample (G1) as a reference, and the X-axes show chromosomal coordinates in kilobases. Deleted ARSs are labeled with grey arrows and ARSs that remained intact are labeled with black arrows. The red bar below *7ori*Δ, near left telomere, denotes the region that has high homology to other chromosomes. Therefore, the elevated BrdU signal at this region is likely due to cross-hybridization with DNA fragments corresponding to other chromosomal loci.

To determine how replication of the *7ori*Δ chromosome VI is accomplished with diminished ORC localization at origins, we performed a replication profiling analysis using bromodeoxyuridine (BrdU) [20] in the presence of HU (Fig. 3). The results revealed significant contribution of *ARS602.5* and *ARS603.1,* which have been recently identified as functional origins [5], [24], to the replication of chromosome VI in both wild-type and *7ori*Δ strains. The distinct peaks of replication at *ARS602.5* and *ARS603.1* in wild-type cells in the presence of HU are consistent with a report by Knott et al, who also used the BrdU-IP method [24]. However, these peaks were much less robust in reports that used DNA copy number-based methods [4], [30]. The basis for the difference is currently unknown. In addition, we unexpectedly detected robust BrdU signals around most of the origins that were deleted. This result suggests that low levels of residual ORC bound around deleted origins are sufficient to support initiation of DNA replication. This almost certainly contributes to the robust growth of the *7ori*Δ strain, and to our knowledge, has never been reported before.

**Figure 4:**
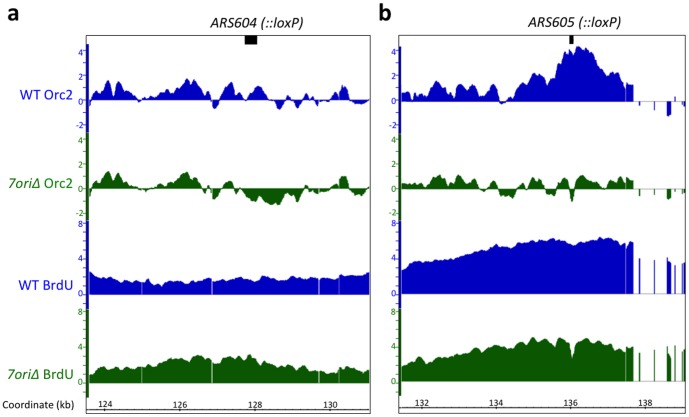
Deleted origins display Orc2 depletion and maintain robust BrdU incorporation. Alignment of Orc2 enrichment and BrdU incorporation in wild-type (blue) and *7ori*Δ (green) cells at (a) *ARS604* (*ars604::loxP in 7ori*Δ) and (b) *ARS605* (*ars605::loxP* in *7ori*Δ). Black bar denotes ARS sequences.

To more closely examine the correlations between the ORC binding pattern and origin activity, we aligned the Orc2 ChIP signals and BrdU replication profiles around deleted origins (Fig. 4). This revealed that replication of the *7ori*Δ chromosome VI initiates at multiple loci near deleted origins, where ORC is bound at low levels. Importantly, we did not observe any detectable increase in Orc2 ChIP signals around deleted origins in the *7ori*Δ strain, suggesting that the background levels of ORC binding seen in wild-type cells is sufficient for origin firing when canonical origins fail. The robust growth of the *7ori*Δ mutant, even in the presence of replication inhibitors, demonstrates that initiation of replication from these sites is sufficient for efficient S phase progression.

Interestingly, the origin activity around the deleted *ARS606* was dependent on the *ADE2* marker, as no origin firing was detected when *ADE2* was replaced with the *KANMX* marker (data not shown). This is consistent with a recent report that a point mutation in ARS606 largely diminishes replication initiation from this origin [31]. The *ADE2* gene on the pRS402 plasmid, which was used as a template for generating a PCR fragment to replace *ARS606*, contains a partial fragment of *ARS1516*, although the predicted ACS sequence was not included [32], [33]. It is therefore likely that the sequence around ARS1516, in the absence of the ACS sequence, contributed to the origin firing at this locus. However, this does not explain the origin activity around deleted *ARS603, 604* and *605*, which have a 34 base pair loxP sequence as a replacement. Because loxP sequence does not have noticeable sequence homology with the ACS sequence, it is unlikely that a loxP site alone can act as an ARS. These results collectively suggest that DNA sequences surrounding the endogenous origins *ARS603, 604, 605* and *1516* have an innate ability to support origin firing. It was previously shown that deletion of multiple origins from disomic *S. cerevisiae* chromosome III allowed proper replication and segregation of this chromosome 97% of the time [10]. It is possible that *de novo* origin firing similar to what we observed contributed to these results as well. Our results suggest that, although replication normally initiates from discrete sites in *S. cerevisiae*, initiation site selection of DNA replication can be unexpectedly flexible in budding yeast, and that yeast chromosomes are capable of initiating replication from non-canonical sites when multiple origins fail.
